# Regaining autonomy, competence, and relatedness: Experiences from two Shared Reading groups for people diagnosed with cancer

**DOI:** 10.3389/fpsyg.2022.1017166

**Published:** 2022-11-01

**Authors:** Tine Riis Andersen

**Affiliations:** ^1^Norwegian Centre for Reading Education and Research, University of Stavanger, Stavanger, Norway; ^2^Faculty of Education, University of Trnava, Trnava, Slovakia

**Keywords:** Shared Reading, cancer patients, arts in health, literature, quality of life, psychosocial intervention, mental health, self-determination theory (SDT)

## Abstract

This study explored 12 cancer patients’ experiences from participating in an online and on-site Shared Reading group for 16 weeks in Norway. Shared Reading is a practice in which prose and poetry are read aloud in small parts and discussed along the way. The study is a qualitative evaluation study with a particular focus on how the participants experienced the reading group supported their life living with cancer. The study was mainly based on the data collected from focus group discussions with the participants, which was analysed qualitatively through open coding. In total, four themes were identified: (1) open space, (2) disconnecting through connecting, (3) community, and (4) resonances and echoes. The participants expressed that the RG helped them to “balance life and cancer”, and “disconnect” from their illness. The cognitive effort needed was beneficial for the participants as a form for “cognitive training.” Since many of the participants had, due to their illness, completely stopped reading books, the reading group also brought literature back into the participants’ lives. Furthermore, it was essential for the participants to feel they contributed to a community, to feel useful and valuable for others. The texts were also important, as some of them resonated strongly with the participants in the way of activating memories and connecting a text to own experiences. After a session, a text could still have an impact as an echo. The results are synthesised, discussed, and supported through the framework of self-determination theory and, more specifically, the basic psychological need theory. The reading group was experienced as a support for autonomy, competence, and relatedness and promoted a feeling of intrinsic motivation that brought about new dimensions in the participants’ lives. The study wishes to increase our knowledge of the benefits of integrating Shared Reading groups as a low-cost, literature-based psychosocial support in cancer organisations.

## Introduction

This study investigated Shared Reading (SR) experiences of 12 patients with cancer, on-site and online, with focus on whether SR can be an alternative way to cope with cancer.

Cancer diagnoses and treatments can have a wide-ranging impact on mental health and the overall quality of life (Walker et al., 2013; Caruso et al., 2017; Pitman et al., 2018; O’Hea et al., 2020). However, the mental health needs of people with cancer are often given less attention during and after cancer treatment, which is mainly focused on treating physical health symptoms and side effects (Niedzwiedz et al., 2019). Thanks to the advances in early detection and treatment, a chronic illness has become an illness people are living longer with; prolongment of patients’ lives are now followed by an increasing recognition of the quality of life of people with cancer (Galway et al., 2012, p. 4). In addition, studies show that the COVID-19 pandemic has caused a high prevalence of psychological problems and gaps in mental health services for cancer patients (CPs) worldwide (Wang et al., 2020; Gallagher et al., 2021). These developments point to an urgency of paying more attention to psychosocial and holistic aspects of health care (Adler and Page, 2008). If the psychological needs are not met, then there is a higher risk for patients to develop mental disorders such as depression and anxiety, which negatively affect the process of recovery (Gordon et al., 2011).

Psychosocial interventions, such as social support groups, have been investigated as an effective way to reduce psychological distress in CPs and improving patients’ quality of life (Osborn et al., 2006; Galway et al., 2012; Sheinfeld Gorin et al., 2012). Within these, there is a major increase of research in the interdisciplinary fields of Health//Medical Humanities, arts in health and Narrative Medicine into the effect of the arts on health and wellbeing. In 2019, this development was recognised in a scoping review published by World Health Organization (Fancourt and Finn, 2019), which showed a robust impact of the arts on both mental and physical health. It identified how the arts can provide a holistic lens to view conditions that are often treated primarily as physical. Although the review includes over 3,000 studies, there are very few studies conducted with fiction reading as most studies with reading are bibliotherapy using self-help books. This does not necessarily indicate a gap in the report, but reflects the fields in general: literary-based interventions have received less attention. This oversight of literature is more problematic when one considers a body of literature within the newly established field of SR that has explored the link between reading and health research. SR is a specific literary activity contextualised in reading groups, developed, and practiced within the UK-based Get Into Reading (GIR) program and in the charity organisation (The Reader, n.d.). It is characterised by reading aloud a short prose text and a poem, a “Reader Leader” (RL) to guide the conversation, and group interaction. SR is a non-clinical intervention, as it has focus on the engagement with the literature and not a pre-determined therapeutic target (Billington et al., 2013). The health outcomes have been tested on various patient groups and have showed positive results for people with dementia (Longden et al., 2016), chronic pain (Billington et al., 2014, 2017; Ohlsson et al., 2018), and neurological conditions (Robinson, 2008b). Apart from those studies, the main body of the research concerns mental health issues (Robinson, 2008a; Billington et al., 2010; Dowrick et al., 2012; Steenberg, 2014; Gray et al., 2016; Ellis et al., 2019; Billington, 2020; Kristensen et al., 2020; Christiansen, 2021; Christiansen and Dalsgard, 2021), and this research is very promising. However, somatic diseases such as cancer, which is associated with heightened risk of common mental disorders, (Zhu et al., 2017), are underrepresented in the current studies.

The purpose of the current study was 3-folds: First, to investigate how SR was experienced as beneficial for people living with cancer. Second, to develop a coherent theoretical framework that captures the complexity of the participants’ experiences, and that can be used as a basis for future research within SR. Third, a more long-term purpose is to move cancer organisations and policymakers to action.

The qualitative and inductive analysis was guided by the language of the participants. A theory that springs from the empirical material is brought into the discussion to frame the findings: Self-determination theory (SDT; Deci and Ryan, 2015) with focus on basic psychological needs theory (Ryan and Deci, 2017).

## Materials and methods

### The organisation of the reading groups

The SR groups were run in two parallel modes in the period September 2021–January 2022 in Norway: one series of physical meetings taking place at a cancer organisation, and one series of online meetings hosted by a hospital library using the secured video platform, Whereby. The participants in both groups had the option to continue for another 4 weeks (16 weeks in total).

The organisation of the reading group (RG) follows The Reader’s SR practice (Davis, 2009; Billington et al., 2013; The Reader, 2019), with 1-h reading and discussing a prose text, often a short story; 30-min reading and discussing a poem. The participants did not read the text beforehand and were not informed about which texts they were going to read. As such, there was no prior preparation required by the participants.

The texts were read aloud by a RL. In the on-site group, the participants received a paper copy, whereas in the online group, the text was shared on the screen. For some of the texts, the RL chose to remove the author’s name to avoid personal bias.

During the reading, the RL initiated pauses that opened for a group discussion. As such, they were discussing the text before knowing how it might continue. Moreover, the RLs and I agreed on some adjustments specific for the target group to avoid fatigue and concentration issues, e.g., a 5-min break between the short story and the poem, using short stories with only few pages, and using more chronological stories. In addition, the RLs sometimes reread the first paragraph, as the participants often needed some time to get into the text, and when discussing, to ensure everyone understood the text, the RLs used a lot of time on “the concrete level”: when, where, what, and who (refer to Appendix 1 for a full description of an on-site and online SR session).

### The Reader Leaders

The RLs were librarians with a Norwegian certification and experience in SR.

### Participants

In total, 12 female CPs consented to participate in the study (eight participants in the on-site group and four in the online group). The mean age was 51 years, the youngest participant being 23 and the oldest 69. The majority, eight participants, had completed higher education, three had vocational degrees, and one a high school degree. Now they worked either part time, were on sick leave, or had retired.

The participants were diagnosed with various types of cancer between 2012 and 2021, and for some of them, the cancer had recurred recently. Therefore, some of the participants were undergoing chemotherapy or other types of cancer treatment, others just finished their treatment, and some were on life-prolonging medication.

The recruitment for the on-site group was *via* local cancer organisations and cancer nurses at the local university hospital, while recruitment for the online group was through national cancer organisations, where the RG was advertised on their social media platforms. For both groups, project information sheets were distributed at cancer organisations, hospitals, and libraries.

The selection criteria for the participants were adults (18+) with a cancer diagnosis, who could understand Norwegian. The intention was not for the group to be all female, although it could be because a RG might appeal more to women than men (Hartley, 2002; Sedo, 2003), and that women in general utilise community offers, for example, the local library, more than men (Applegate, 2008).

The attendance rate was highest in the on-site group with two to six (out of six) participants in each session. In the online group, there was less stability in attendance with one to three (out of four) participants in each session. The attendance rate was lower because some participants had treatment and surgery in between. During the course of the RGs, two participants withdrew from the study: one from the on-site group and one from the online group.

### Data collection

Data were collected during 16 reading sessions from the two RGs (*N* = 12), over a period of 4 months.

#### Data collected during group sessions

In the beginning, the participants filled out a background questionnaire to collect demographic data, information on the participants’ cancer diagnosis, reading habits, and motivation for signing up. During the sessions, I collected data through participant observation (Fangen, 2017) in 31 sessions supported by field notes and audio recordings. The benefit of using this method is to get an in-depth experience and understanding of the participants’ experiences and the phenomenon studied.

#### Data collected after the reading group

The intervention was evaluated in a final focus group session of approximately 2 h with the participants, one for each group, and an additional focus group after a 4-week SR extension with three new participants in the on-site group. The focus groups started with a 5-min prompted writing task: 1) What has been most important for you in the RG? (refer to Appendix 2 for the interview-guide). Then, a semi-structured interview was conducted with the RLs to get further insight into how SR can be adapted to CPs.

### Data analysis

The analysis was inductive, and data-driven, which is why I decided to use open coding on the transcripts of the focus groups. First, I read and reread the transcripts, and during the second reading, I did a pre-coding (Layder, 1998) by commenting and highlighting everything of interest. Then, I continued with a structural coding in NVivo, also known as “utilitarian coding” (Namey et al., 2008; Guest et al., 2012), to code data segments into the different elements in the RG (e.g., group discussion, reading aloud, the RL, the participants, the texts, or outcomes). Hence, the similarly coded segments were grouped, and I could go into the content of the individual structural codes and continue analysing in-depth. At that point, I used a descriptive coding, combined with *in vivo*-coding (Glaser and Strauss, 2010, reprint; Charmaz, 2014; Corbin and Strauss, 2015), two coding strategies that are close to the terms and language used by the participants. Thus, I worked with the data on two levels: on a structural level and a more analytic level. Afterward, I continued the coding process manually, sorting the references to the participants’ experiences from the focus groups into categories. This manual process, after the coding process in NVivo, helped me to do an axial coding by grouping the codes into bigger categories and then into bigger themes. In this process, I worked with mind-maps and discussed my codes and categories with peers, and these steps helped me to see overlaps and connections in the material. I ended up with four overall themes, which present the essence of the data. Together, the themes constitute a theory enhancing our understanding of why SR is experienced as beneficial, and how it can potentially work as a coping mechanism for CPs. I went back and forth between data and my interpretations in an iterative process.

### Reflexivity

#### Preunderstanding

Before the data collection, I took the course to become a RL in Norway to get a richer understanding of SR both as participant and RL. I was in general aware of previous and ongoing research within SR.

#### Role as a researcher and a participant

The participants were informed beforehand about my presence in the sessions as observer *and* participant.

I found it difficult to write field notes during the session, as I was also a participant and had to follow the reading and the discussion very thoroughly. Also, it might have been experienced as a disruption for the participants; if I were taking notes all the time, it could have added to their sense of being observed.

I experienced a dilemma coming from the two positions in “participant-observation”; my research interest would sometimes influence my participation. I will explain it through an empirical example: We read the poem “The Road Not Taken” by Robert Frost (2019) and talked about choices in life and how our choices had led us to where we are today. Halfway through the discussion, I said: “It can also be an event that brings you on a new road.” Without saying the word “illness,” I still had the link in my mind, and the participant Alice followed up on it: “Or when you become sick.” I am not sure if the conversation would have, by itself, without me interfering, gone that direction, but it turned out to be an engaging discussion about how they felt cancer limited them in their lives but also had led to new choices (new roads). Since I was also a participant, it would be difficult not to impact the conversation at all, but these experiences made me more aware of my different roles.

#### Lens of the participants

A preprint was sent to the participants for member checking and feedback (Korstjens and Moser, 2018).

## Results

In the qualitative analysis, and in my categorisation and interpretation of the data, four overall themes emerged. The themes each cover a dimension of the participants’ experiences of what they regarded as important for them in the RG. The themes are as follows:

• *Open space*: The experience of a safe environment which is conducive to a process of self-expansion
• *Disconnecting through connecting*: The experience of forgetting worries for a moment by focusing on the engagement with the text.
• *Community*: The experience of contributing and being in a collaborative social environment with other CPs.
• *Resonances and echoes*: The experience of the participants’ felt connection to the text and the group. After a reading session the resonances become “echoes.”

Together, these themes can be structured in a proposed theoretical model suggesting a theory grounded in the data, on how SR might work as a supportive environment for CPs. I am using the concept of theory from an interpretivist perspective informed by Lincoln and Lynham (2011). In a constructivist paradigm, theory building is a descriptive activity, and the purpose is to provide deep understanding of the lived experience of participants by the researcher (Lincoln and Guba, 1985; Lynham, 2002). The proposed theoretical framework in this study is captured and explained through a model to visualise how, based on my interpretation, the themes are connected as a whole and placed in the context of a SR session^1^.

### Process model of the overall themes

**FIGURE 1:**
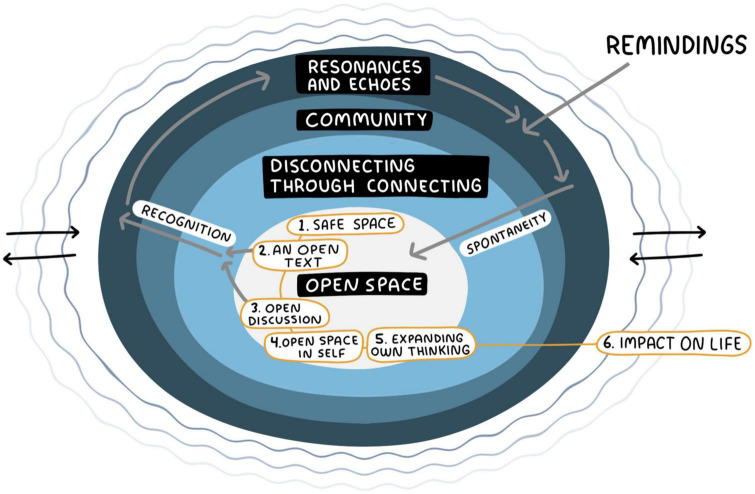
Process model of Shared Reading.

The model presented below (refer to Figure 1) outlines the processes underlying SR sessions and how these impact participants’ lives and that of people around them. Central for these processes is a network of agents: the texts, the participants, the setting, and the RL.

The model is not hierarchical but includes four central elements that influence one another: the environment, the engagement with the text (reading and listening), the group, and the stimuli (the texts and personal stories). In the middle is the *open space*, which contains the components on which the RG is built on – the core. In the open space, there is a process connected to the dynamic in a SR session with reading and discussing a text together (see the yellow boxes). The process of self-expansion shows how (1) a perceived safe space in a reading session and (2) the meeting with an open text, likely will lead to (3) an open discussion sharing different perspectives, which can then create an (4) open space in oneself, by embracing others’ ideas and perspectives, and as a result (5) expand one’s own thinking, which might in some cases (6) impact the participants’ life outside the group.

The grey arrows in the model that begin from “2. An open text” and “3. open discussion” shows how the text, or something another participant says, can create resonances in the participants through recognition. The resonances can then trigger remindings in the individual participant, for example, a memory, which comes from outside the RG space in the participants’ personal lives, and are brought into the group. Some of them are shared in the open space through a personal story, which adds spontaneity to the open space. After a reading session, some of the resonances might turn into what I call echoes, that is when the text or the reading experience keep echoing in the participants’ lives. The echoes are illustrated in the model as ripples. The black arrows are a way to show that the echoes do not disappear, but are brought to the next session, and in that way, the sessions build on each other and create an environment for transformation.

I will in the following sections elaborate on each of the four themes and the different components in the model.

### Open space

I will start with the model’s core – the *open space*. This theme is divided in three closely related categories: “safe space,” “balance between life and cancer,” and “process of self-expansion.”

#### Safe space

The participants referred to the RG as a place where they could talk about the text, but also about anything in particular, including sharing personal stories. The participants could be themselves, in other words, sharing and talking freely, able to express own ideas and be comfortable enough to share different perspectives in the group. This was possible as there was a respectful tone in the group, communicating that every input is welcome. Furthermore, I observed in the sessions that the participants made room for and listened to each other; for example, when a participant talked for a longer time during the group discussion, the person was normally not interrupted by the others, not even when there were longer breaks in the talking where the participant seemed to think and search for the right words.

One of the participants described the atmosphere with the following words: “And then there is no fact list. There is room for… Just as in life there is room for everything. No matter what we mean and think and feel then everything is allowed, right.” (Susanne, on-site focus group, 106:34)

In the model, “safe space” is presented as the first step in the process of self-expansion. In this section, I will go further into the category to present how and why the participants perceived the RG as “safe.” The concept of safe space, where “everything is allowed,” was constructed of several components that intertwine and overlap and that I have grouped in four subcategories:

##### Mutual understanding

It was crucial for the participants that the group was only for CPs as there was a mutual understanding coming from a shared embodied experience of living with cancer. Social activities and meetings with the “outside,” for example, following a conversation and interacting in social settings where people are not aware of their cancer diagnosis, was in general considered very tiring and challenging for the participants. For the same reason, they were hesitant about participating in a public RG at the library. They were worried that it would give them this feeling of incompetence they sometimes have in their social life:

Because the head is cotton, right. I am dangling around and have to perform everywhere, right. When people don’t know it [that she has cancer], I think it can be… That it can be challenging for me right. Because they don’t see… Like they can’t see it on us. (Amber, on-site focus group, 11:50).

The quote points to the necessity that the reading group was targeted to CPs, because it provided a safe space that functioned as a contrast to the “outside.”

##### Low threshold for participation

The participants emphasised the significance of how the group was organised and facilitated (for an elaborated characterisation of SR see Dowrick et al., 2012). In SR, there is no preparation for the participants, no pressure to say anything, and listening is also valued as a part of active participation. In addition, it is a core value in SR that it is the participants’ experience of a text that is foregrounded, and not an academic approach (Billington et al., 2013). Although the RLs introduced the SR concept in the beginning of the RGs, it was only when the participants experienced it in practice that they really grasped it, when the RL did not have the “final answers” to the text, and when the participants’ input to the text was met and recognised as valuable contributions. This freedom to say anything, or just listen, took the external pressure off the participants.

The participants also said that they appreciated the consideration that the RL and I showed them by adjusting the SR practice to their shared needs and challenges, which gave them more flexibility in their participation. Moreover, the on-site group found it helpful that the RG took place at the local cancer organisation, which was a place where they felt comfortable.

In the RG, there were other expectations than in a public RG, as the participant Rachel expressed:

I would have wanted to be in a normal reading group, but it wouldn’t have worked (…). Like my well-being is changing a lot. And here I have in a way the opportunity (…) You can participate in a part of it, you can participate and be a bit passive if you don’t have the energy. (Rachel, online focus group: 49:00)

Ease of participation helped them to take part on their own terms and it gave them a sense of volition.

##### The Reader Leader’s engagement

The participants said it was a central factor for them that the RL showed a personal engagement to the texts and also shared from her own life, as it made it easier for them to share as well. The positive feedback from the RL was emphasised as especially important for creating a pleasant atmosphere in which the participants felt comfortable to say something: “She responds to everything in a very nice and caring way. And she also shared from her private life which makes it feel more safe for me” (Marta, on-site group, 43:00). It was also important that the RL had a warm, caring, and open presence, being more in line with “the one who brings the literature,” or a co-participant, instead of a “leader.”

##### Recognition and distance through a literary text

The text can also be considered an essential component in the construction of a safe space through the combination of distance and recognition. One of the participants explained that when she was reading about someone else, there was a distance to herself, that helped her to open emotionally, and it eased some personal issues she was dealing with. Although the story was about someone else, it was also a bit about her. Thus, the texts provided an indirect channel into feelings and reflections about parts of the participants’ illness and life in general. Reading a variety of literary texts that did not directly address cancer, but touched on universal feelings, such as, loneliness, love, wonder, sadness, and themes and situations they could recognise in their own lives, from past experiences or through someone they knew, opened naturally for sharing personal stories. The text functioned as a catalyst, or “holding ground” (Davis, 2020) for thinking and feeling, and the text was in some way the foundation of the group, providing a base for interesting conversations about life, as one of the participants explained:

Well, it has been surprising how great it has been for me [to be in the reading group]. (…) But it wasn’t really because of the texts. But without the texts there wouldn’t have been… Right, the text is the basis, that influences the whole group in a way. (Susanne, on-site focus group, 34:26)

The conversations and interactions in the group happened *through* and due to the presence of a literary text.

#### Balance of life and cancer

These four elements combined: “Mutual understanding,” “low threshold for participation,” “the Reader Leader’s engagement,” and “recognition and distance through a literary text” provided a safe environment for the participants, where they were able to balance life and cancer, which can be seen as the function of the open space: “We are in an illness process and then we do something that is not about that. And that actually gives me strength. Mmh.” (Lisa, online group: 48:33).

In an interview with one of the RL’s, she explained that the participants did not come to the RG as patients, but with their “healthy side”. This was possible since the groups did not have a direct focus on the participants’ cancer. Additionally, it was because of this experience, of doing something else in an illness process, that the participants experienced the RG as a special arena where there was room for their past experiences *and* their present situation. In the group, they could talk about cancer *and* about other things, a setting which they said was new for them. They explained that other activities specifically offered to CPs were either related to and circling around cancer or not related at all with little room for talking about their illness. A SR study with chronic pain patients (Billington et al., 2017) found a potential therapeutic effect in the recall of *life* experiences, not merely experiences of pain/illness, helping to recover a whole person and not just an ill one. This finding resonates well with the participants’ experience of balancing life and cancer, which also had an aspect of perceived autonomy as the participants decided for themselves when and how much they wanted to share.

The participants talked about their cancer story as something they had with them in the RG, and that came to the surface when the text gave language or imagery to reflect on and express their illness experiences. Thus, the sharing of cancer happened more naturally than in other settings where there is a therapeutic target, e.g., in a group therapy session. Moreover, the RG provided a space without close relatives and generated new topics for conversations with friends and family which were not about their illness. This was experienced as a relief for the participants because they felt it was sometimes heavy and sad to talk about their cancer with relatives or friends. In the RG, talking about cancer was easier as it was with “strangers,” which helped them to talk more freely about heavier topics. Since the RG was only for CPs, they explained, they could go beyond the topic of cancer and be themselves.

#### Process of self-expansion

In the process of analysing the data, open space seemed to be conducive to a process of self-expansion and change (see the model on page 5). In the focus groups, the participants talked about intra- and interpersonal processes that emerged from a safe environment. For example, they became more aware of their own and others’ way of being and thinking. The participants’ increased awareness and acceptance of multiple perspectives was overall regarded as a valuable experience. One of the participants, Amber, explained it the following way:

…I don’t have the truth on things here. Like, my way of understanding things is one thing, but you understand it in a completely different way, which can be just as correct what you understand, right. I believe it was kind of good for me and when, for example, she said something or you said something then I thought, wow, it can also be understood in that way. And then I started to reflect more and then I could relate to what had been said. (Amber, on-site focus group, 07:00).

The quote touches on the central aspect, which is the realisation that arose when meeting with perspectives that differed from their own and a reflection about how people in general think differently. Although this would seem like something that was not particularly surprising information for the participants, they said they experienced it as an “aha”-moment because the group discussion facilitated the meeting of multiple perspectives in a very concrete and “embodied” way.

In the model, the process of self-expansion is conceptualised as a path with different stages for the purpose of clarity. In practice, it might not be a linear path, and the factors will more likely mutually influence each other in a fluid way.

##### An open text starts an open discussion

In the RG, the participants read and listened to literary texts with multiple voices or perspectives through meeting different “people” and life views. Reading a layered, ambiguous, and often open-ended text, which can be interpreted in several ways, stimulated the participants’ wonder, questions, and imagination: “When there is this kind of [open] end you start to think yourself” (Susanne, on-site group: 96:00).

The open spaces in the texts initiated an open discussion where the participants shared their different views, experiences, and interpretations of the text. The times where the participants became “more embracing” toward others’ way of thinking does not necessarily imply that the participants always agreed with each other, or with the text, but instead, they listened and met each other and the text with generosity.

##### Expanding own thinking

The awareness of multiple perspectives in the room both led to an increased consciousness of the participants’ own way of being/thinking versus others, and in some cases, it also led to a change in their own mind set, or how they understood a text as the others’ input and the text itself kept challenging their thinking and opened their mind for alternative possibilities. This helped one of the participants with breaking negative or restricted patterns of thinking:

And it touches so important themes in my life at least where I am now, right. Because I sense this dark future, what is that. And then there is the hopeful future. And you stand in between all this, right. And I feel that I can, like, carefully approach this through the texts. And when I interpret the text with you afterward it feels more safe to do, because… well, it can be understood that way or that way. So, in a way I don’t lock myself in my own pattern. (Susanne, on-site focus group, 11:41).

The dark and the hopeful future is related to an uncertainty in her prognosis, but instead of going into her own pattern of thinking, the discussion opened for approaching “the dark future” in new ways.

##### Impact on life

The experience of becoming more tolerant, embracing, and generous as a person by listening to the other participants happened temporarily during the RG, but they also talked about being conscious about the other participants’ ways of thinking in other settings of life, asking if a situation/text could be understood in a different way: “Like for me it is something I take with me to other situations as well, these voices from the other participants. This about things do not necessarily need to be that way, they can also be in another way” (Rachel, online focus group, 48:00). In that way, these repeated meetings, or confrontations, with other perspectives, accepting and sometimes incorporating them in their own way of understanding themselves and others, supported a self-expansion:

I have always been the one who had the answer to things, right, I have probably irritated a lot of people in life. And I think. Yes [laughing] I have. But I come here, sit with you all, I listen to you. Then I become like more embracing [“romslig” in Norwegian]. Because I understand that I can’t just insist that I read things my way, right. And that has probably impacted my daily life as well, actually… (Susanne, on-site focus group, 4:09).

This process of self-expansion was experienced as something the participants learned from the RG, something they had trained and had become better at.

### Disconnecting through connecting

Around the open space is the second theme I identified: *disconnecting through connecting.* Focusing on engaging with the texts, by listening to *and* reading short stories or poems, helped the participants to forget the current concerns they had on their mind: “I think, what is nice here, by coming here, is that you get a free minute, right. That you forget everything in a way. And you go into the texts, and that is where you have focus, right.” (Amber, on-site focus group, 21:00).

Previous SR studies has mainly focused on the “soothing” and relaxing effect of being read to (see for example: Dowrick et al., 2012, p. 17; Billington et al., 2010, p. 43), where less attention has been giving to the benefits of the parallel activities of listening *and* reading. Skjerdingstad and Tangerås (2019) describe it as a “double modality” in which “affords the recipient easier attention to the work” (p. 6). The combination of reading and listening involves two different but overlapping reading experiences: First, the participants were experiencing the text mediated through the RL. Second, they could follow the text themselves either on print or on screen. In the observation of the groups, I noticed during the sessions that most of the participants followed the text themselves. Sometimes an outburst of laughter or surprise arrived before the RL had reached the place in the text, which showed that the participants from time to time were ahead of the RL. I also observed that “two of the participants only followed the text partially, and the rest of the time they closed their eyes, looked to the side or out of the window” (Field note, session 1). The fact that the participants could follow the text themselves seemed to give them an element of self-control and autonomy in the reading. It also happened often that the participants became “co-facilitators” by pointing out and reading out loud a passage they found interesting and wanted to bring to the group’s attention.

These parallel activities, reading and listening, and the combination of them are what constitute the second theme which contains the categories: “cognitive training,” “a richer reading experience,” and “being here and now.”

#### Cognitive training

The participants pointed out in the focus groups that they “activated” their minds in the RG as they had to focus to be able to follow the story: “So sometimes she has read aloud and many times I’ve thought I should tell her to repeat, because I can’t… It was just as if I mixed things all wrong, and thought that’s because my head isn’t ready.” (Elena, on-site focus group 24.21)

The aspect of required focus in listening and reading had a beneficial outcome for the participants’ as “cognitive training.” The activity of reading aloud supported the activity of reading, as the participants did not have to read the text themselves; to have the text in hand or on screen was equally experienced as a great aid, a kind of anchor to follow the reading aloud. As many of them struggled with maintaining concentration and had severe headaches, reading small parts together every week helped them to slowly get back into reading.

During the RG, several participants went from not reading at all, despite identifying as readers, to starting to read entire books again because they experienced their ability to concentrate had improved. In some cases, this was correlated with a reduction in side effects from the cancer treatment, and general health recovery, but the RG as a structured activity for cognitive training might have boosted this improvement. As the participant, Molly explained:

…and this is the only type of structured activity, I participate in, where I challenge myself on what I struggle with the most, that is the cognitive. I have to. understand, to remember things, concentrate on something. (Molly, online focus group, 83:29).

Additional reading, engagement, and training cognitive capacity were in some cases naturally related to each other, because when the participants improved their ability to concentrate, it became easier for them to read more, and the experience of being engaged in a text motivated them to read more.

In one case, the SR form was experienced as too demanding due to severe side effects of multiple rounds of treatment. Alice, from the on-site group, was not able to follow the group discussion, when people were talking at the same time, or keeping her concentration while listening to the reading aloud. She said that the treatments “stays with you” (while saying it she pointed to her head). For her, it was easier with the poetry, as a poem consists of fewer words, often limited to one page, and thereby easier to get an overview of. In general, the participants perceived the short stories as more demanding, in terms of concentration, than the poems which the participants described as “the easy one” and a “fresh change” after the short story. This finding seem to differ from a large body of SR research where “the story appeared to foster relaxation and calm, while the poem encouraged focused attention” (Dowrick et al., 2012, p. 17).

#### A richer reading experience

It was not only a matter of being able to read again, but also about re-experiencing a love of reading or a re-appreciation of the literature. One of the participants was not able to remember very much from when she was reading alone because of cognitive impairment, and her reading experience was as such very poor:

If I try to read a book, then I use an awful lot of time on it and I feel I lose so much and therefore I have to read the book from the beginning again. I remember very little 2 days after I have finished the book. While here I feel I get more out of it. Because it is read aloud and then I can see it myself. That I can read at the same time. So that combination is good for me. (Rachel, online focus group, 19.56).

It was not the reading aloud in itself that helped her in the RG, but the combination of reading and listening, because she also struggled to keep focus when listening to audio books. The slow pace in reading aloud was also an important factor. The slowing down made it easier for the participants to get into the text, because they would notice and remember more things, and they said that their experience of the texts in the RG was “deeper” than when they read themselves. One of the participants emphasised the fact that every word in the text is read aloud in SR, but when she reads herself, she would often skim some passages, for example, nature descriptions. This made the experience of the text in the RG more absorbing. Moreover, the RL introduced the participants to new literature and dwelled at places they would not necessarily do themselves, which made them aware of other aspects of the text and of literary texts in general, for example, focusing on and going in depth with a beautifully written passage or unfolding an image in the text. These instances of dwelling made the experience richer and surprising, because they would never know where the text, the RL, and/or the other participants would bring them. The participants also believed that discussing a text with others gave the text “another dimension.” One of the participants expressed how stunned she was that the group could get so much out of a couple of pages. She pointed out that if she had read one of the short stories or poems herself, she would have read it very quickly and not reflect much about it or go in-depth with single sentences as they did in the RG. Because they only read a short story and a poem, and often a story of couple of pages only, this absorbed and focused their attention while reading and discussing the text.

#### “Being here and now”

Another element of going into the story or poem is “being here and now.” The participants describe it as coming to the RG with an open mind without having to prepare anything, being served a text – a literary experience, be entertained and get a break from their self; from “performing” in social settings and an everyday preoccupation with cancer treatment. This experience of being present in the moment and not having to worry or think ahead acted as stress relief and mindfulness:

Well sometimes you can analyse the room like also when you are in different settings, but here I have not done that. I have just felt that it was great to be here and deal with things when they arrived (.) Well, I don’t have any homework or anything, I just have to connect and then I have to practice this [to be here and now] (Molly, online focus group, 29:55)

The participant Lisa explained in an interview that it was not only about being deeply engaged in something else, but also with *someone* else. Through the short stories and poems, and especially the short stories, the participants were invited into fictional people’s lives and problems, focusing on the feelings and dilemmas presented in the text. Thus, it was easier for them to forget the heavy and sad thoughts on their mind and delve into the complex life of another person. Lisa described this experience as being “lifted up” and “out” of herself, and she felt it was a relief for her to forget herself for 2 h, go deep into the text and focus on something (or someone) else than her cancer, e.g., going into another time époque, or in a different life perspective. As such, “being here and now” does not necessarily entail to be in the present time, but to be flexible and transcend to where the story and the RG brings them.

The category is also related to the slow pace in the reading aloud. It was experienced as a contrast to “the outside,” and to how they act in other settings, for example, at work or in social life. The participants emphasised that, due to the slow tempo, the RG, was not efficient at all. This was a challenge for some of them as they were used to seeing themselves as an “efficient” person in the way that they had an urge to go faster through the text. However, they felt it was good for them to challenge this part of themselves, practicing to slow down, dwell and be more present in the moment:

“One of my trademarks before I got ill was that I was very effective [“effektiv” in Norwegian]. And this here [the RG] is not effective, it takes… it has its pace and there is no right or wrong answer, right. It challenges me in many ways and in my way of being for many years. And that is what I like so much about it, and I believe is so good for me. To try to slow down, be here and now, be open, think there is no right or wrong” (Rachel, online focus group: 43.10)

The interrelated categories of “Cognitive training,” “a richer experience,” and “being here and now” represent different dimensions of the participants’ experiences related to the engagement with the texts. The activity of listening and reading helped the participants to stay focused and personally involved which led to them becoming more immersed in the story and disconnecting.

### Community

The third theme is community; when there is a strong sense of community, it is more likely that personal stories are shared in the open space and that the participants can disconnect. The participants had a strong need for belonging because many of the participants found it difficult to maintain social relations due to fatigue, and to communicate their illness experiences to friends and family. Although many of them had a supporting social network, they talked about “being alone in their cancer,” a feeling of loneliness that some of them had experienced for the first time in their life.

Community contains the categories “to do something meaningful for yourself with others,” “contribution and collaboration,” and “social relations.”

#### To do something meaningful for yourself with others

Many of the participants were on sick leave, retired, or working reduced hours, a life situation which can be very challenging, they explained, as you have little structure in life and a lot of “free-time”: “There is no difference whether it is Monday or Friday, right, you don’t get this Friday-feeling which is one of the good things in life, a break because it is weekend…” (Bobbi, on-site focus group – 91:08). The RG helped the participants by adding a rhythm to the week, occupying the day, getting out of the house and receiving fresh input from others. In general, doing something which they prioritised and regarded as meaningful. The participants explained it as “choosing their own medication,” which gave them a feeling of agency.

The participants explained that being part of something they felt was important and meaningful motivated them. The RG was something they chose to prioritise, and although it was tiring sometimes, it also gave them energy being there.

#### Contribution and collaboration

The participants talked about another aspect of how cancer changed their life; a sense of uselessness came from situations of not being able to contribute as much at work, at home and in life in general as they did before they got ill. Regaining value was put forth as central to the participants:

Now I work 30% this autumn and earlier I partially haven’t worked at all. This thing, to feel important for someone (…). I feel that you [the other participants] have communicated that to me, that I am important in this group and to know that has been very import in my life. To have a thing in my daily life where I still have significance, because at my work… Well, I have a workstation and some tasks, but I am not important in the same way as when I worked full time (.). So, in that way the group has been very… Meant a lot for my illness and recovery. (Rachel, online focus group, 62:42)

The aspect about feeling valuable and valued was related to the positive feedback from the RL and the other participants in the group. The RL responded to the participants’ input in the group discussion, recognising them as competent contributions by responding with phrases such as: “oh, that was very interesting,” “thanks for sharing that with us,” and by referring to something a participant had said: “I really liked what Susanne said earlier about the….” Feedback was also related to their presence in the group and was expressed in the sessions, but also in follow-up e-mails addressed to each participant after a session or shortly before the next as a gentle reminder. This feedback before and after a session was important for them as it was adding to their feeling of being appreciated and valued.

The participants in general felt a strong need for sharing. This sharing happened in both groups, but where more frequently in the on-site group, as they had more opportunity for small talk and got to know each other better which led to sharing more. The participants shared tips about hikes, book recommendations, memories, illness experiences, coping strategies and advice, tears and laughter, frustration, irritation, feelings, and perspectives across generations. This act of sharing strengthened the community feeling.

Furthermore, it was also essential for the participants that it was an interactive and cooperative group where the individual participant’s contribution helped the group to open and understand the text. If just one of the participants saw something in the text, and shared it with the group, the other participants could follow along and maybe, although they did not understand or liked the text themselves, resonate with the other participants’ views on the text and build on that. This was an essential dynamic in the group and can be interpreted as the participants were “opening doors” for each other in the text which kept the discussion going. The participants also talked about experiencing the text together at the same pace. In general, when they discussed a place in the text, they talked about it as though they had been there together. In the focus group, Marta tried to recall the memory of a specific text: “That house, do you remember? Then we came home to it, [and] we didn’t know if it was the mother-in-law or…” (Marta, on-site focus group, 41:00).

The experience of contributing and collaborating in the RG made the participants feel part of something, and they felt competent and useful again.

#### Social relations

I had initially anticipated that the social part was not as important for the online group as they did not meet each other in person, but this was not the case. In the focus group, all the participants mentioned that the social aspect and meeting the other participants had been very important to them. However, in the online group, the socialising happened more through the group discussions and sharing different perspectives on the text, and less through “small talk.” In the on-site group, they were forming new relations and had contact with each other outside the RG. In the online group, they appreciated meeting someone and socialising the 2 h they were there, but they did not get to know each other in the same way, or the process of getting to know each other was slower. In the online group, it seemed to be more about contributing to a community.

Susanne from the on-site group said that each of the participants had become a part of her as “eternal friends.” It was not in the way of frequently seeing each other, but she felt she could always reach out to them if she needed to. Rachel from the online group experienced the social interactions as a “by-product” of the RG; the other participants’ stories, the things they mentioned which were happening in their lives, inspired her and she got curious and engaged in the other participants’ lives.

### Resonances and echoes

#### Resonances through recognition

Moving to the outer circle in the model brings us to the fourth theme: *resonances and echoes*. The theme is related to the personal connections between the individual participants, the group, and the text. A connection could be a feeling, a mood, a memory, or a reflection that the text triggered in the individual and which resonated with them. There were often connections between the participants’ lives and the text that created a resonance in the participants, a form for *recognition* or identification of a situation, place, person, attitude, feeling, or belief in the text. It was not necessarily an identification with a story character, as the participants did not believe they identified very strongly with the characters they met in the different texts during the RG. Identification was instead an experience they understood and related to when reading a whole novel or watching a TV series over a longer period. Instead, they talked about the recognition in the text, and that the text had to trigger their interest or a personal story. This included, for example, reading a story that took place in the same surroundings as where they grew up, or reading about lonely women who stayed home during the day, a theme one of the participants related strongly to because of her own situation of being at home when others had daily routines such as work or school. They also talked about recognising a feeling in the text, to which they could relate and identify, e.g., the feeling of loneliness, the feeling of waiting for an answer, of being in love, or preparing for the worst. One of these moments of recognition happened when we read the poem “Betring” [recovery] by Gyrðir Elìasson (Elíasson, 2016). The poem has 27 words and is about a person who puts down a torch on a rampart an autumn evening, surrounded by darkness, and walks slowly into the light. The participant Rachel recognised her own experience of recovery in the poem’s images:

“It is not a full lighting you walk into, it is not super sharp or clear and maybe a bit unclear boundary between the torch light and… Like the beam from the torch and the darkness around. And the dew also makes it a bit like maybe more unclear and that it is like not a very… It is not a straight line when you recover from something. Well, that is my experience, right. So, I think that was a very good image.” (Rachel, session 7, online group, 01:49).

The poem offered her an image that she, and the other participants could explore and expand by placing herself in the poem (use of third person singular: “*you* walk into”). The poem gave her language, or images, to reflect on some aspects of recovery. In that way, you could say she was reflecting and maybe understanding better her own experience through the poem, and at the same time, she approached and understood the poem through her own experience of recovery.

The participants did not necessarily know what touched them beforehand, but the texts that resonated with them were experienced as significant. Some resonances were shared with the other participants in the “open space” through a personal story or reminding.

#### Remindings

Remindings, associations, memories from the participants’ lives, and past experiences were activated by the text or during the group discussion and brought to the present time – to the RG. In the model, remindings are visualised as a stimuli coming from “outside” the RG. They are uncontrolled and bring spontaneity to the open space: “Like, suddenly something comes from someone and then it starts a thinking-process in us, or feelings, and then we talk about it…” (Alice, on-site focus group, 89:38).

Considering the type of memories that were shared during the sessions, the short stories seemed to trigger memories connected more to childhood and growing up – to the past. Whereas the poems seemed to trigger reflections and experiences from their present situation. This might be due to the more narrative form in the short stories, and that the participants believed the poems “had a bigger room for interpretation” as they touched on more universal and existential questions and feelings.

Nevertheless, the poems could also activate memories; for example, when reading the poem “Romanske buer” by the Swedish poet, Tomas Transtrømer (2011), the participant Elena experienced “entering” a memory from a tough time when she experienced stress. The poem’s description of inside a church reminded her about an experience she had had herself in a church during this period. She explained that, during the reading aloud of the poem, the church and her memory appeared as an image in her mind, and she felt the church from her past was “in front” of her. She also said that she started to remember more details of the memory during the reading session, some of which she had forgotten about. The memory reminded her about going through something tough and overcoming it. It made her realise that her cancer, because she was getting better, also would become just one part of her life story that she could look back on.

These memories from the participants’ past lives, which were brought to the present, were important for them, as it was a means to go through the personal processes related to them, and then let them go. One of the participants explained this process as “cleaning up” the past.

#### Echoes and ripples

After a reading session, the text or the reading experience could still have an impact on the participants. This I have interpreted as “echoes.” The participant Molly talked, for example, about her experience of how the poem “Konkylie” by Olav H. Hauge (1951) kept “coming back to her mind” in the weeks after reading it in the RG: “That one [the poem] I felt still long time after. I remember bringing it up under the dinner saying to my husband that now you have to listen… right. And I started to tell, read, and it really stayed with me. The poem kept like coming back again and again” (Molly, online focus group, 58:39). She describes the poem as it has almost a will of its own. This poem was from the first session, but despite her troubles with memory, it is the text she remembered best from the RG. It was especially important to her because it reminded her to “to put a time limit to sadness.” She also started to write about the poem. Thus, the echoes of the poem led to actions and small transformations.

In the model, “echoes” are placed in the outer circle together with resonances, but they also continue after a reading group session. Echoes can disappear when leaving the room/logging out of the meeting. They can also linger for a longer time or disappear and then return. An example of a delayed echo, activated by the surroundings, could be when the participant Lisa sent me an e-mail months after the RG had ended with a picture of herself in a meadow of reeds, smiling. The place had reminded her about a short story we had read, where a girl was running through very tall reeds. She got inspired to run through them herself and felt she was experiencing the same as the girl in the story. She said, being taken back to the story, gave her energy and inspiration, and she asked her friends to take a picture of her to capture the moment.

Echoes can also be reinforced and become stronger again by keeping the text/reading experience alive by telling others about it, rereading or keeping on reflecting, which can create new echoes. Some of the participants googled the author when they came home from the RG. One of the participants explained that she did it not only to get more information about the text, but to understand better the “heart” behind the text – which experiences it originated from. In these ways, the texts kept echoing in the participants. Apart from the echoes of the texts, the RG also seemed to have a “ripple effect” over time where the whole experience of being part of the RG, meeting the other participants, the RL, the researcher (me), and the texts, impacted them and created ripples that spread to their lives and the lives of the people around them: “It is like that butterfly effect or ripple effect; you start doing something and then it becomes big in your life (…) it has done so much” (Susanne on-site group, interview).

The continued reading experience, conceptualised here as an echo, points to the idea that the time between a reading session is not empty. When a text, and/or reading experience, keep echoing, a lot can happen within the individual participant. The echoes are brought back to the next session, and in that way, it is a circular and upbuilding process, which can create big or small ripples in the participants’ lives.

## Discussion

### Relations between the four themes

This study explored the experiences from a RG with CPs and how it supported their life with cancer. Based on the findings, one can conceptualise the processes unfolding in a SR session as follows: It was through the feeling of a safe environment, that the participants could balance life and cancer and a process of self-expansion seemed to take place. The open space can be seen as the foundation of the RG, as it helped the participants to engage in SR (connect and disconnect) and to feel comfortable enough to share personal stories, activated through the texts, which strengthened a sense of community.

“Disconnecting through connecting” is related to the engagement with the texts. Through cognitive engagement (connecting), and a way of engaging with the text which was experienced as “richer,” the participants’ attention was moved to an experience of “being here and now,” which helped them to disconnect. The participants seemed to go back and forward between connecting and disconnecting, showing how the process is not necessarily unidirectional. This finding, that a cognitive effort was needed, seems to differ from other SR studies where an atmosphere created by the reading offers “a centre toward which participants can gravitate” (Longden et al., 2015, p. 115). The experience of “disconnecting through connecting” might be similar to how chronic pain patients experienced forgetting their pain, momentarily through engagement with complex and cognitive demanding texts (Billington et al., 2014). I have further investigated how CPs bodily discomforts and cognitive challenges interacts and are supported by a “double modality” – with reading aloud in a slow tempo combined with reading themselves.

The existence of a strong community surrounds the participants’ engagement with the texts. Community is built on the experience of doing something meaningful for themselves with others, contributing and collaborating, and by meeting other people with cancer. Community strengthens the other components in the model (more personal stories are being shared and it is easier for the participants to disconnect). Moreover, discussing the text together helped the participants to get into the text, to “connect.” Community played an important part in my study, which most likely was because the group was for CPs where the mutual understanding made it easier to share and relax.

Surrounding the community is the resonances and echoes within the individual participant. The theme has both an individual and collective dimension; the experience of recognition activated individual remindings from an entire lifespan. Some were shared spontaneously in the open space which led the discussion in new directions and the participants continued to build on what was shared – a form of “collective reading experience.” After the RG, some of the resonances turn into “echoes,” which are brought back to the next session, so the model becomes circular and dynamic. Therefore, a SR group probably needs to run for some time before it might have a more lasting impact on the participants lives.

The themes are closely related, and it is the combination of them that makes SR a potential coping mechanism for CPs.

### How can Shared Reading support cancer patients’ need for autonomy, competence, and relatedness?

In the process of understanding what might be going on in the data, I found the self-determination theory to be central to the participants’ experiences, regaining a feeling of contribution and usefulness, feeling related and a part of a community, and overall do something they prioritised, on their premises which brought intrinsic motivation.

#### Self-determination theory and basic psychological needs

The self-determination theory (SDT) is a psychological and empirically based theory about motivation, addressing “what energises people’s behaviour and moves them into action” (Deci and Ryan, 2015, p. 486). SDT proposes that all human beings have three basic psychological needs (BPN) – the need for *autonomy*, *competence*, and *relatedness.* In SDT, needs are defined as “nutrients that are essential for growth, integrity and wellbeing” (Ryan and Deci, 2017, p. 10). Moreover, a social environment can either “provide the nutrients for growth” (needs are supported) or “disrupt and impair the process” (needs are thwarted; Deci and Ryan, 2015, p. 487). *Autonomy* refers to an individual’s perceived agency, and the need is satisfied when we feel we are the origin of our actions, and our behaviour has a sense of volition. *Competence* is the experience of mastery and of operating effectively within important life contexts. *Relatedness* is the need to feel connected with others, to feel understood and cared for, but also to feel significant among others, and equally important, to experience oneself as giving or contributing to others. Relatedness is therefore both directed toward close others, but also to a sense of being part of social organisations and social groups (Ryan and Deci, 2017, p. 11). SDT also differentiates between different types of motivations: *controlled motivation* and *autonomous motivation* (Deci and Ryan, 2015). A controlled motivation is driven by external factors and has an element of obligation, seduction, or force, whereas an autonomous motivation is characterised by choice, enjoyment, and voluntariness. Intrinsic motivation, which is an internal and natural motivation, is the prototype of autonomous motivation. Researchers have proposed that satisfaction of the three needs leads to improved mental health (e.g., lower depression, anxiety, and higher quality of life) and in physical health-related outcomes such as eating healthier, physical activity, and improved adherence to prescribed medications (Ryan et al., 2008).

A fourth need in SDT is suggested by Slater et al. (2014) through the theory of “Temporarily Expanding the Boundaries of the Self” (TEBOTS). Slater et al. propose that our core motivation to engage with narratives is to get a temporary relief from the task of maintenance, defence, and regulation of the personal and social self. Moreover, they suggest that by reading fiction, the boundaries of self are expanded, and the reader is not limited to rules of social norms or morality. In addition, the theory hypothesises that to disconnect in a “profound way,” you need to be immersed in a story. This is exemplified by the act of reading while on holiday; we may escape daily pressures by relaxing, but we need a book to take us mentally away to expand the self (Slater et al., 2014, p. 444). Thus, the TEBOTS-model also indicates a possible relation between “open space” and “disconnecting through connecting,” between engagement and self-expansion.

The findings in this study broaden the TEBOTS theory by including mechanism in SR that facilitates immersion that is not the property of the narrative: multimodal, pauses, discussions, and re-readings. Moreover, TEBOTS are exclusively referring to narratives, but the participants could be immersed in a narrative as well as a poem.

#### A cancer diagnosis thwarts basic psychological needs

Perceived autonomy is important regardless of health status, but particularly important in the context of a serious chronic illness because the illness limits the individual’s autonomy. Furthermore, when people are diagnosed with serious illnesses, such as cancer, their learned knowledge and behaviour might not be sufficient, and this presents challenges to their feeling of competence (Ng et al., 2012). Cancer can be experienced as quite invasive and autonomy limiting; it often requires radical lifestyle changes, it can impact work, social and personal life negatively, and individuals have little control over how their illness progresses. Specifically, during treatment, cancer is likely to reduce autonomy and patients might particularly benefit from autonomy support (Cosme and Berkman, 2018).

A study interviewed colorectal CPs during adjuvant treatment and found that CPs often experience BPN frustration when undergoing cancer treatment (Romero-Elías et al., 2021); the patients felt autonomy frustration when not being in control of the negative impact of the treatment on life. Relatedness frustration, as they reported not being able to perform the same social plans, also feeling less connected to their environment, and competence frustration as they felt “useless” from being more dependent on other people, e.g., relatives. The participants in my study also experienced similar frustrations coming from a thwarted BPN. In addition, because of the challenges with concentration and memory the participants’ ability to immerse themselves in a narrative, the need for temporary relief from maintenance of the self was also thwarted.

#### Shared Reading as autonomy, competence, and relatedness support

Discussing the findings in the present study in light of SDT theory, we can say that the RG supported an autonomous motivation in various ways. First, the participants’ participation was on their own terms; they were informed that they could participate to the extent they were able to, and in whatever way they wished, with no pressure to say anything. Second, the RL and I were highly flexible in meeting the participants’ individual needs: there were, for example, no requirements to be present in every session, or an entire session, and there was no homework. The participation was in these ways, self-initiated because, although it was a research project they had signed up for, they experienced the group as something *they* had chosen (“choosing their own medication”) and that *they* prioritised, because they enjoyed being there and it gave them energy. This is the essence of intrinsic motivation.

The autonomy was also supported in the sense that the space the group provided was not limited to talking about their cancer. In addition, the texts were not about cancer either, but brought a wide range of conversation topics related to all aspects of life into the room. At the same time, there was also room for the participants’ cancer, and the mutual understanding made it easy for them to switch between talking about cancer and talking about other things. Hence, they could regulate, and balance life and cancer based on their individual need and the sharing of their cancer story was voluntary. Moreover, autonomy was supported through the participants’ role as “co-facilitators”; they were not only listening to the story read aloud, but they engaged in the reading themselves.

The role as a co-facilitator also supported a need for competence. The fact that they presented their own views and thoughts, which sometimes differed from the other participants and the RL, indicated self-confidence. Moreover, by sharing their own views, they experienced how the others listened attentively to their contribution, taking it seriously, and responding with positive feedback. These experiences further supported their perceived self-confidence and competence.

In SDT, the need for competence refers to “a feeling of being effective in life.” The slow pace of the reading sessions challenged the participants’ learned expectations of needing to be and act efficiently. This was regarded as a positive experience, helping them to enjoy a setting in which they did not need to meet expectations (neither their own nor others’), which they could not live up to since they got ill. Thus, they got a break from these external and internal expectations by engaging in a slow activity, dwelling and being present in the moment, and this was something they valued as a richer and more rewarding experience. One of the participants experienced to be “lifted up and out of herself” (see page 10), which perhaps can be explained as a need for relief from expectations and from defence of the self as proposed in the TEBOTS model. Hence, you might say that the RG supported their need for competence but challenged their need for being effective. Thus, the experience of competence when you have a cancer diagnosis might nuance and change the need for competence as described in SDT.

Although one of the participants, Marta, did not participate much in the group discussion, but after the first two sessions, something changed in her; she started to become more active and was the first to respond and openly showed when she disliked a text. Marta’s big change in the RG might be because she had a thwarted need for relatedness when she joined the group, as she was experiencing relatedness frustration in her social relations. Marta felt that her friends did not understand her illness situation, as none of them had cancer themselves. They had difficulties in understanding that, although she had finished her treatment, she still did not feel well, but was actually feeling worse. It was difficult for her in general to manage social life because she did not have the energy to be active. She had experienced her social network becoming smaller. This difficulty of maintaining social relations was also recognisable for the other participants; being part of the SR community supported their need for relatedness. Furthermore, relatedness was supported through literary recognition, from “meeting” different types of “people” in various life situations. Some of the texts strongly reflected a feeling of the participants, e.g., loneliness or being in nature, and as such, they felt connected to some of the texts. We were, for example, reading a short story about a boy who crawled up a honey suckle plant, and the participants remembered the times they had encountered its strong smell. One of the participants also said she could recognise and almost feel the pain of the branches in her hands.

The RG, and the texts, echoed and rippled in the participants’ lives – it “energised” the participants’ behaviour and “moved them into action” (see quote on page 13). The participants started to read more, were inspired to write poems and autobiography, and continued the discussion of the texts with friends or families. In an interview, the participant, Susanne, said that she had a strong wish to share her experience of SR with others. She was very eager to set up a SR group in her local community.

The participant Alice, who experienced the SR form being too demanding and therefore preferred the poem, seemed to be experiencing competence frustration. During the RG, she was often pointing to her limitations and challenges, saying “she was lost in the text,” “she was tired,” “she didn’t understand anything,” and “her head was not working anymore.” For the remainder of the session, she would often change from focusing on the text to using the group as a room for venting of frustrations connected to her illness experience. She participated in four sessions, and although she was challenged in the activities, she expressed that the social part in the RG had been important for her. Thus, she particularly benefitted from relatedness support and autonomy by being able to vent frustrations and express her emotions in a safe environment. But her experience of competence frustrations might have been what kept her from continuing in the group. Alice’s experience points to a group of CPs that might be too challenged by cognitive impairment to engage in SR.

### Which findings are specific for cancer patients?

The findings and the model are to a great extent not limited to CPs. Although, I can only speculate about which findings are specific for CPs and which are “core” experiences in SR. I believe the framework can be transferred to a general SR group, but “community” and “disconnecting through connecting” could be more specific for CPs as they can have a more pertinent need for relatedness and competence. The participant Elena told me that she afterward joined a SR group at the local library. She said the biggest difference was that fewer personal stories were shared in the group. Since it was an open drop-in group, she did not experience either being part of a community. Therefore, the experience of being part and contributing to a community based on unspoken mutual understanding might be essential in SR groups for CPs and in general for people with illness. Moreover, the participants seemed to have a strong need for disconnecting, for cognitive training, and to have an open space that functioned as a contrast to “the outside” where they could get a break from being “efficient” and balance life and cancer. This points to the SDT being more relevant for CPs, or people with illness, where the model and TEBOTS might could hold for all participants in SR.

### Recommendation for future research

A meta-analysis of self-determination theory applied to health contexts (Ng et al., 2012) examined the empirical literature testing SDT in health care and health-promoting settings. Overall, the meta-analysis supported the value of SDT as a conceptual framework to study motivational processes and, importantly, to plan interventions for improved health care, improved well-being and quality of life. The study also found that autonomy support in health care positively predicted higher levels of autonomy, competence, and relatedness. In light of these findings, I propose SDT as a framework to facilitate and study SR’s potential link to mental health. Future research could further investigate the BPN fulfilment in SR groups, and the occurrence of indications of need fulfilment, as a help to predict mental health outcomes. The following questions remain to be answered: Is the fulfilment of needs dependent on medium or on target group? Does it differ per text genre or text content? Is the need fulfilment in SR phenomenologically different from other types of support groups, for example, psychotherapy, or other artforms? What is the role of the text? Could, for example, the experience of a safe space alone be enough to have an impact? And how might SR be improved to foster and facilitate the BPN? Future research could also investigate in more depth the continuous impact of the texts and the RG in the time between a reading session, and methodical differences between online and on-site SR groups, which has not been part of the present papers’ purpose.

The number of participants of the current study is very small, which obviously affects the general conclusions that can be drawn from it. It would be beneficial to the research and the theoretical framework to implement a larger study, extending the number of SR groups (both in person and online) for CPs, also including men. It would also be a valuable contribution to incorporate video recordings, to capture bodily gestures and facial expressions, which might add nuances to the findings. Moreover, the study did not triangulate the data used for this paper with audio recordings of SR sessions due to practical reasons of time constraints. In a future study, I aim to explore the theme “resonances and echoes” in depth by analysing the transcripts from RG sessions. This might further adjust the theoretical framework and the model presented in this paper.

## Conclusion

The objective of the current study was to investigate SR as an alternative, low-cost psychosocial offer in a clinical setting, or online, and its benefits for CPs evaluated through the patients’ experiences. The findings from the study have demonstrated that SR was experienced as a supportive environment that fulfilled basic psychological needs. By drawing on perspectives from SDT, the study has presented a plausible explanation for how and why SR contributes to mental well-being. Apart from answering the question about why SR “works” in a comprehensive way, the study also provides a reliable framework from which other researchers can generate new research questions.

## Data availability statement

The raw data supporting the conclusions of this article will be made available by the authors, without undue reservation.

## Ethics statement

The studies involving human participants were reviewed and approved by the Norwegian Research Ethics Review Board. The patients/participants provided their written informed consent to participate in this study. The names that are used in this article are fictitious.

## Author contributions

The author confirms being the sole contributor of this work and has approved it for publication.

## Footnotes

1 Centre for Research into Literature, Reading and Society (CRILS), in collaboration with The Reader, has previously developed a suggestion for a theoretical model of SR: “Theory of change” (Davis et al., 2016). The theory is based on the idea of feeling a “stuckness” in life and how SR, by a change in perception, can help move people out of their “stuckness”. The model is therefore more related to general well-being, where the situation is slighly different from people who are diagnosed with a possible life-threatening illness, such as cancer, that can disrupt the normal track of life. The diagram proposed in this paper is not a “theory of change” but a process model of Shared Reading.

## Abbreviations

BPN: basic psychological needs
CP: cancer patient
RG: reading group
RL: Reader Leader
SDT: self-determination theory
SR: Shared Reading.
