# Supplementary material for: Regaining autonomy, competence, and relatedness: Experiences from two Shared Reading groups for people diagnosed with cancer
Source: Front Psychol. 2022 Nov 1;13:1017166. doi: 10.3389/fpsyg.2022.1017166 (PMC9665161; doi:10.3389/fpsyg.2022.1017166)
Supplement: Supplementary file 1 [file Data_Sheet_1.pdf]

Appendix 1:

DESCRIPTION OF AN ON-SITE AND ONLINE SHARED READING SESSION:

Description of an on-site reading session:

Wednesdays between Sep. 2021-Feb. 2022 at 14.30-16.30 I went to a local cancer organisation in Norway. The organisation is voluntary driven by funding and the patients do not pay any kind of membership. It is a place where cancer patients can take a break and meet up with others. The organisation also offers different courses and physical therapy sessions.

When I arrived, there was always someone from the staff to greet me and the participants and to offer us a cup of coffee. The reading group session took place when the host organisation was closing, so we had the room to ourselves and there were no disturbances or other people listening. The room was cosy, with plants, decorations, couches, and a kitchen where we could get coffee or tea whenever we wanted. Due to COVID-19 regulations of social distances we were sitting two meters apart on chairs placed in a circle, which means that we had to use the whole room. Later, from session five onwards, we moved to a sofa arrangement in the corner of the room, and the distance between the participants was changed to one meter, in line with new national regulations. From this meeting onwards, I noticed that the atmosphere became warmer and cosier, as we were sitting more comfortably with two tables in the middle, that connected us.

Given the difficult circumstances with the ongoing pandemic, we were very lucky that we could carry out the reading group, as there were in general very few activities because of COVID-19 restrictions. The participants very much appreciated coming out and meeting people. As we were very few people, maximum seven people in the room, it was possible.

From the very beginning of the reading group, there was a lot of small talk, before and after the group and in the breaks. The host organisation usually provided some baked goods for us, or one of the participants, me included, had brought sweets or munchies. Everyone was sitting comfortably with coffee and cake when the Reader Leader sent the short story for today's session around and, when everyone had the text in front of them, started to read. Her voice was soft and pleasant to listen to and when she started to read, the text began to live – she gave it life, intonation, tempo, and atmosphere. The Reader Leader usually made between two or three stops during the reading, often followed by a long silence, but it was not an empty silence, but a time for thinking/feeling, and usually when it happened the participants looked down at the text, turned the pages to skim what had been read. Then the Reader Leader broke the silence with an opening question to start the discussion, for example: "Do you have any thoughts about what we have read?"

After the short story, the Reader Leader read a poem aloud twice, and the participants talked about it. After the Shared Reading, the participants were asked to 'empty their head' on a piece of paper, meaning that they wrote for 10 minutes. There were no requirements or instructions for what they

should write about, beyond whatever thoughts, ideas, and associations the texts and the discussions had evoked. Some of the participants would evaluate today's session, others would write about a memory activated by the texts, or the discussions, others would write some keywords they found important for the session or the text. There were no right or wrong way to do this. However, as it was research and the participants' texts were collected afterwards, some of them were in a bit of doubt about what I wanted. I emphasised after some sessions that they could write about something personal, which helped them feel freer in their writing. The activity was completely voluntary. After the writing, if there was time and the participants had the energy and motivation, they answered a questionnaire to both the short story and the poem. If there was no time left, the participants were encouraged to complete the questionnaires at home before next session. Some of the participants completed them just before the following session, others did it the day after, both with the consequence that their answers were not as 'fresh' as they would have been if completing the questionnaires after the readings.

A description of an online reading session:

The structure of the online group was similar to the on-site group with one hour including breaks for group discussion for the short story, and 30 min. for the poem, but there were also some differences. In the online group, the text is shared by the Reader Leader on the screen. The participants can still follow the text, but they do not have control over it in the sense that they cannot turn the page themselves, go back, reread. Instead, they are dependent on the Reader Leader scrolling in the text.

In the online group the participants could choose if they wanted to have their camera on, and if they wanted to be muted. When the participants were muted, the spontaneity of their responses during reading was lost. When they were muted, it also seemed to require more for them to unmute and speak. The implication was that small, spontaneous responses were not articulated in the same way as in an on-site group and the group discussion was less natural and less 'flowing'. Instead, they mostly said something if they had something specific to say. They spoke often for a longer time, and their input was more focused with a clear intention and target. The participants usually had their camera on, but some turned it off under the reading aloud. They were also unmuted most of the time, but because of technical problems with echo in the sound, they had to mute sometimes.

In the online group there was not much small talk, and our aim of being there was the texts. In a five minutes break, usually between the short story and the poem, the participants often went to get some food or a cup of coffee/tea. Very few times did anyone talk with each other in the break, and we simply waited for the Reader Leader to start the session again. I was unsure, in my role as the researcher, if I should have started a conversation, because maybe they needed to have complete silence in this small break.

Another difference between the physical group and the online group was that the participants were joining from their homes, or at the hospital during their chemotherapy, or another place, which made the participation in the online group very flexible and adapted to the participants schedules and needs.
