# Supplementary material for: Regaining autonomy, competence, and relatedness: Experiences from two Shared Reading groups for people diagnosed with cancer
Source: Front Psychol. 2022 Nov 1;13:1017166. doi: 10.3389/fpsyg.2022.1017166 (PMC9665161; doi:10.3389/fpsyg.2022.1017166)
Supplement: Supplementary file 2 [file Data_Sheet_2.pdf]

Appendix 2:

Interview guide: focus groups

Focus group evaluation of reading groups for people with cancer
(after 12th session and after 16th session)

Table over focus groups:

Focus group	Time	Attendance	Duration
1: onsite	After 12th session	N=4 (4)	2 h
2: onsite	After 16th session	N= 3 (6)<br>3 new P and 2 from the original group)	2 h
3: online	After 12th session	N = (3)	1.5 h

Purpose and focus:

1) The participants' experiences of the different parts of the reading group (the texts, the reading aloud, the group discussions, the writing, the research part, the context, the social)
2) Possible impact on life
3) The experience of participating in a reading group in an illness process

Note: The interview guide is a broad collection of questions related to the three purposes described above. This is only a guide made beforehand, and not the actual questions asked. The focus group discussions did not include all of the questions, although most of them got covered in the participants answers. The questions were asked in this particular order either (although they did start with the introduction) I also asked follow up questions to what the participants chose to focus on. The questions asked in each of the focus groups varied as it depended on which way the discussion went.

Introduction (5 min. Individual writing):

- What has been most important to you in the reading group?
- What do you feel you got from the reading group? (what are you taking with you)

Reading aloud

- How did you experience to listen to the texts read aloud?
- How did you experience reading a story in parts?
- I noticed that some of you were sometimes only listening to the text, will you say a bit about that? How did you experience only listening?
- How did the rest of you experience listening *and* reading yourself?

The reader leader

- How did you experience the reader leader?

The other participants/the social

- How was it to meet the other participants?

- How has it been to be in a reading group with other cancer patients?

Group discussions
- How did you do experience the group discussions?
- How did you experience listening to other people's perspectives/ide's/personal experiences related to the texts?
- How did you experience the discussion about the short stories?
- How did you experience the discussion about the poems?

The texts
- Is there a text you remember especially well? (why that one?)
    - How did you experience reading the text?
- Were there any texts you didn't like? (why?)
- How did you experience the texts that did not have a clear ending? (specific for the on-site group)
- (How did you experience reading and speaking of texts about illness? --> no texts in on-site group that dealt with illness
- Did you read any of the texts again?
- Do you still think about some of the texts?

The writing (only for on-site group)
- How did you experience the writing?
- What do you think about writing after reading a text and talking about it?
- What did you write about?

Research:
- How did you find that someone observed and participated in the reading group at the same time?
- How was it to fill out questionnaires along the way, to be recorded, and keeping the audio diary?

Context:
- Location: What do you think about the reading group being held at Vardesenteret?
- Time: What do you think of the time of the reading group? (morning/day/afternoon)
- Duration: 12 sessions in two hours. Is it too small/too much/appropriate?
- How did you experience the different room setups? First that we should sit on chairs two meters distance and then sofa arrangement?
- How has it been to participate in the reading group during COVID-19?

Possible impact:
- Did the reading group impact you in any way? (how?)
- Did your illness have a role in your motivation for participating in the reading group?
- Was there something you wanted to get out of the reading group? Did it fulfill it?
- Do you think the reading group helped you somehow in relation to your cancer? (if so, can you mention some examples on how it helped you?)
- Do you read more books/literature than you did before?
- Did you start something new during the reading group?

- Can you mention a specific thing or situation that you believe the reading group has impacted?

Extra questions related to reading:

- Did the reading group impact your reading habits? Do you read more/less?
- Do you read in a different way? Do you notice other things when you read?
- Do you read other books/texts than you did before?
- How do you experience reading at home/reading together with others?

Questions specific for online-reading group:

- How did you experience to participate in an online reading group?
- How was it to be in a reading group with people you haven't met in real life?
- How was it to share something personal
- What was the good thing about an online reading group?
- What didn't work?
- How was the atmosphere?
- Would you prefer a physical or online reading group? (why?)

Last questions:

- What do you think about a reading group as an offer in cancer organisations?
  - If you compare the reading group to other activities, courses, you have been participating in, do you think the experience was similar to them or did it differ in a way? How?
